# First report of gastric endoscopic intermuscular dissection

**DOI:** 10.1055/a-2233-2914

**Published:** 2024-03-07

**Authors:** Edward J. Despott, Laura A. Lucaciu, Alberto Murino, Alessandro Rimondi, Kenneth Binmoeller

**Affiliations:** 1 171090 Royal Free Unit for Endoscopy, The Royal Free Hospital, University College London Institute for Liver and Digestive Health, London, United Kingdom of Great Britain and Northern Ireland; 2 7153 Interventional Endoscopy, California Pacific Medical Center, San Francisco, United States

Although endoscopic intermuscular dissection (EID) has been used successfully to treat rectal pathology 1 , its application in stomach procedures has not been reported. EID involves the dissection of the circular layer from the longitudinal layer of the muscularis propria to achieve a clear vertical dissection margin 2 3 .

A 43-year-old woman was referred to our center for endoscopic resection of a 15-mm type 1 neuroendocrine tumor (NET) located in the lower gastric body ( Fig. 1 ). Saline immersion therapeutic endoscopy-facilitated EID was performed under general anesthesia ( Video 1 ). A gastroscope (GIF-1TH-190; Olympus, Tokyo, Japan) with a water jet was used for saline exchange and aspiration of bubbles or gas. EID was performed using a 1.5-mm ball-tip knife (FlushKnife BTS; Fujifilm, Tokyo, Japan) and a prototype partially circumferential cap attachment, designed to provide tissue counter-traction, a wide endoscopic field of view, and facilitate instrument passage without friction (Micro-tech, Nanjing, China) ( Video 1 ). Despite previous endoscopic ultrasound having shown the NET to be entirely submucosal, our procedure revealed it to be adherent to the muscularis propria, necessitating EID to achieve a tumor-free deep resection margin. The resection site was closed in two layers using through-the-scope clips (Resolution Clip; Boston Scientific, Natick, Massachusetts, USA). Histopathological analysis confirmed the tumor to be a type 1 gastric NET, arising on a background of chronic atrophic gastritis with enterochromaffin-like cell hyperplasia ( Fig. 2 , Fig. 3 ), with no lymphovascular or perineural invasion (World Health Organization Grade 2), and an R0 resection was achieved. Following our protocol, the patient was discharged after 72 hours without any adverse events.

**Fig. 1:**
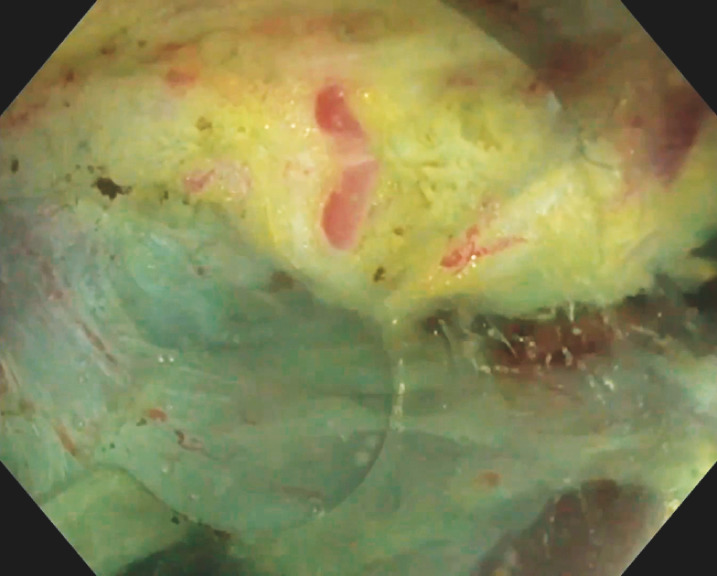
Neuroendocrine tumor adherent to the inner oblique layer of the muscularis propria.

Saline immersion therapeutic endoscopy–facilitated endoscopic intermuscular dissection (EID) of a type 1 neuroendocrine tumor (NET) located in the lower gastric body.Video 1

**Fig. 2:**
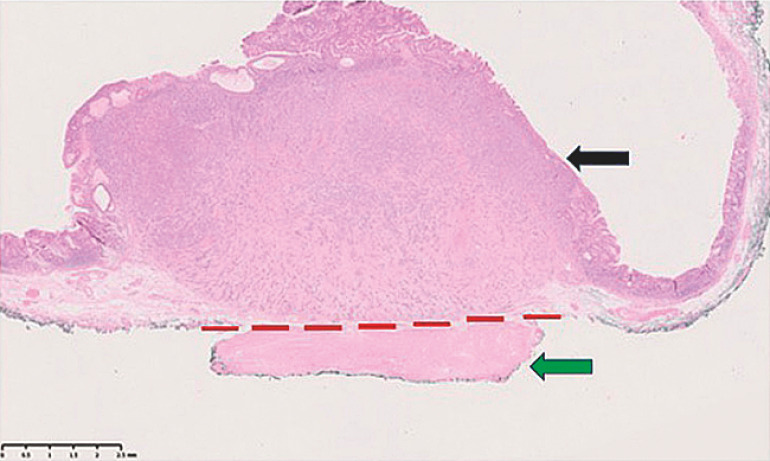
Histopathological analysis of hematoxylin and eosin-stained section demonstrating a grade 2 neuroendocrine tumor (black arrow). The green arrow indicates the inner oblique layer of gastric muscularis propria. Dissection only above the interrupted red line would have resulted in an R1 (noncurative) resection.

**Fig. 3:**
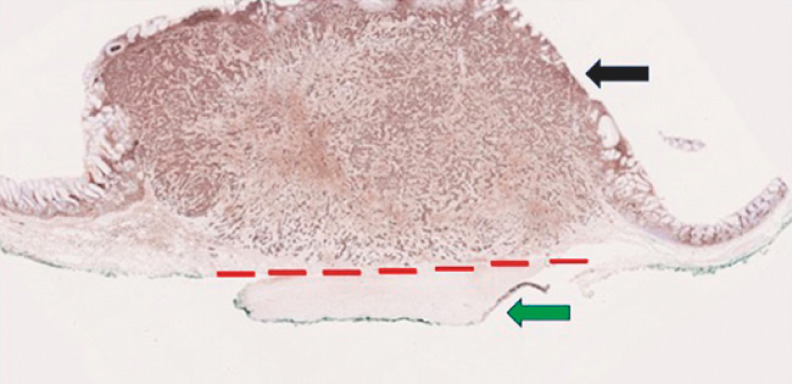
Histopathological analysis of chromogranin-stained section demonstrating a grade 2 neuroendocrine tumor (black arrow) invading the inner oblique layer of the muscularis propria (green arrow). Dissection above the interrupted red line would have resulted in an R1 (non-curative) resection.

This first report of gastric EID demonstrates the feasibility of this technique for gastric pathology when deeper excision margins are required to achieve R0 resection. Further studies on the wider application of gastric EID in clinical practice would be worthwhile. Furthermore, the use of saline immersion therapeutic endoscopy is crucial for ensuring clear visualization and buoyancy during EID 4 .

Endoscopy_UCTN_Code_TTT_1AO_2AG

## Notes

Endoscopy E-Videos https://eref.thieme.de/e-videos
*E-Videos* is an open access online section of the journal *Endoscopy* , reporting on interesting cases and new techniques in gastroenterological endoscopy. All papers include a high-quality video and are published with a Creative Commons CC-BY license. Endoscopy E-Videos qualify for HINARI discounts and waivers and eligibility is automatically checked during the submission process. We grant 100% waivers to articles whose corresponding authors are based in Group A countries and 50% waivers to those who are based in Group B countries as classified by Research4Life (see: https://www.research4life.org/access/eligibility/ ). This section has its own submission website at https://mc.manuscriptcentral.com/e-videos .
